# Ventilation Management in a Patient with Ventilation–Perfusion Mismatch in the Early Phase of Lung Injury and during the Recovery

**DOI:** 10.3390/jcm13030871

**Published:** 2024-02-02

**Authors:** Ana Cicvarić, Josipa Glavaš Tahtler, Tajana Turk, Sanda Škrinjarić-Cincar, Despoina Koulenti, Nenad Nešković, Mia Edl, Slavica Kvolik

**Affiliations:** 1 Faculty of Medicine, Josip Juraj Strossmayer University of Osijek, 31000 Osijek, Croatia; josipa.glavastahtler@kbco.hr (J.G.T.); turk.tajana@kbco.hr (T.T.); neskovic@mefos.hr (N.N.); medl@mefos.hr (M.E.); 2 Department of Anesthesiology, Resuscitation and Intensive Care, Osijek University Hospital, 31000 Osijek, Croatia; 3 Department of Radiology, Osijek University Hospital, 31000 Osijek, Croatia; 4 Department of Pulmonology, Osijek University Hospital, 31000 Osijek, Croatia; 5 2nd Critical Care Department, Attikon University Hospital, 15772 Athens, Greece; deskogr@yahoo.gr; 6 UQ Centre for Clinical Research, Faculty of Medicine, The University of Queensland, Brisbane 4029, Australia

**Keywords:** ventilation, pulmonary, lung injury, personalized ventilation, hypoxia, pulmonary diffusing capacity, intensive care, pneumonia, *Stenotrophomonas maltophilia*

## Abstract

Chest trauma is one of the most serious and difficult injuries, with various complications that can lead to ventilation–perfusion (V/Q) mismatch and systemic hypoxia. We are presenting a case of a 53-year-old male with no chronic therapy who was admitted to the Intensive Care Unit due to severe respiratory failure after chest trauma. He developed a right-sided pneumothorax, and then a thoracic drain was placed. On admission, the patient was hemodynamically unstable and tachypneic. He was intubated and mechanically ventilated, febrile (38.9 °C) and unconscious. A lung CT showed massive non-ventilated areas, predominantly in the right lung, guiding repeated therapeutic and diagnostic bronchoalveolar lavages. He was ventilated with PEEP of 10 cmH_2_O with a FiO_2_ of 0.6–0.8. Empirical broad-spectrum antimicrobial therapy was immediately initiated. Both high FiO_2_ and moderate PEEP were maintained and adjusted according to the current blood gas values and oxygen saturation. He was weaned from mechanical ventilation, and non-invasive oxygenation was continued. After *Stenotrophomonas maltophilia* was identified and treated with sulfamethoxazole/trimethoprim, a regression of lung infiltrates was observed. In conclusion, both ventilatory and antibiotic therapy were needed to improve the oxygenation and outcome of the patient with *S. maltophilia* pneumonia and V/Q mismatch.

## 1. Introduction

Chest trauma is one of the most serious and difficult injuries. It can occur because of penetrating or blunt trauma and can be life-threatening [1,2]. After trauma, various complications can develop, including the development of pneumonia, hemothorax, pneumothorax, and rib fractures, all of which lead to a ventilation–perfusion (V/Q) mismatch. Ventilation–perfusion mismatch (V/Q) is a serious condition that can lead to systemic hypoxia. It is also the most common cause of hypoxemia. Alveolar units can move from a low V/Q region to a high V/Q region in the case of disease. A low V/Q ratio produces hypoxemia by decreasing alveolar oxygen delivery and decreasing alveolocapillary gas exchange (PaO_2_). Subsequently, the arterial oxygen level will drop. In the presence of a larger area with hyperventilated and less perfused areas of the lung parenchyma or high V/Q units, changes in oxygenation may be so slight as to be unnoticeable. Disturbance of gas exchange can be manifested by an increase in the level of CO_2_ in the systemic circulation, with low end-tidal CO_2_, such as in pulmonary embolism [3]. In these situations, physiologic compensation for V/Q disorder will be a change in the breathing frequency or H_2_CO_3_ levels.

The presence of large areas of non-ventilated yet perfused lung parenchyma is observed in atelectasis. Preventing or reducing non-ventilated lung areas (atelectasis) and rebalancing the pulmonary vascular tone is a challenging therapeutic strategy in the treatment of patients with acute respiratory failure [4]. An important compensatory mechanism during hypoxemia, particularly when chronic, is hypoxic pulmonary vasoconstriction. It is a homeostatic vasomotor mechanism that is intrinsic to the pulmonary vasculature [5,6]. Intrapulmonary arteries constrict in response to alveolar hypoxia, diverting blood to better-oxygenated lung segments, thereby optimizing oxygen uptake in atelectasis, pneumonia, asthma, and adult respiratory distress syndrome. Hypoxic pulmonary vasoconstriction mediates ventilation–perfusion matching and optimizes systemic PaO_2_ by reducing shunt fraction [7].

However, impaired hypoxic pulmonary vasoconstriction, whether due to disease or vasodilator drugs, exacerbates systemic hypoxemia. High perfusion in relation to ventilation (V/Q < 1) and shunting (V/Q = 0) is caused by not only impaired hypoxic pulmonary vasoconstriction but also redistribution of perfusion from obstructed lung vessels [8]. During the causal treatment, this imbalance must be corrected by increasing the percentage of oxygen and lung ventilation pressures, which leads to the correction of hypoxemia.

We will present an approach to the treatment of V/Q mismatch and systemic hypoxemia in acute pulmonary injury after massive lung contusion and during the recovery phase in a 53-year-old male patient admitted to the Intensive Care Unit (ICU) due to severe respiratory insufficiency after chest trauma. We will also show how the late consequences of lung trauma, fibrosis, and bullous lung changes led to an impaired diffusion capacity of the lungs in this patient.

## 2. Case Report

**Figure 1:**
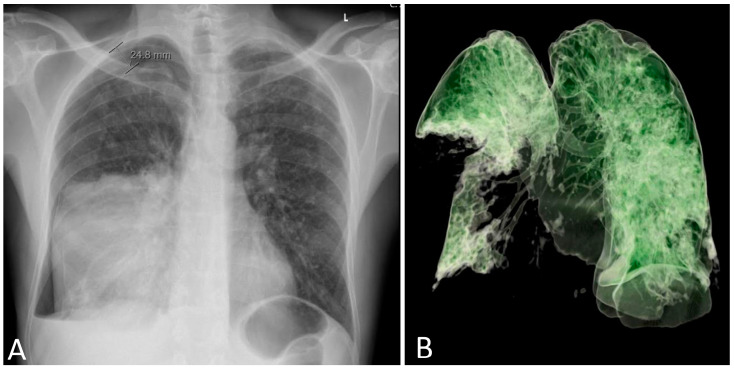
Chest radiography on hospital admission with right-sided pneumothorax and a pulmonary infiltrate of the medial and lower lobe of the right lung (**A**). A complete absence of aeration of the lower and medial right lobe was present on day 3 (**B**). L: left side.

A 53-year-old male patient with no comorbidities, except for being a smoker, without chronic therapy was admitted to the ICU due to severe respiratory failure. Five days before admission, the patient was hit by a metal object on the right side of the chest and complained of shortness of breath. Chest radiography revealed a right-sided pneumothorax, and a thoracic drain was placed (Figure 1A).

**Figure 2:**
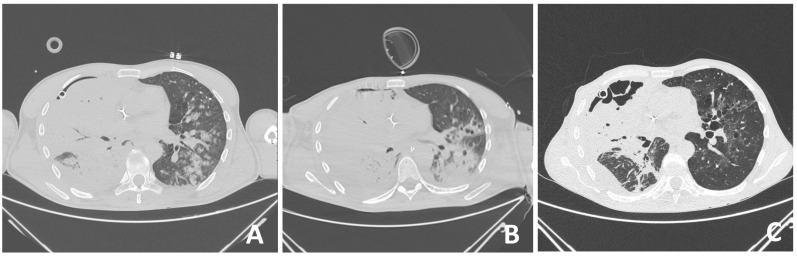
Changes in the lung window of CT in a 53-year-old shipyard worker after severe chest trauma with V/Q mismatch and respiratory failure requiring mechanical ventilation: on admission (**A**), after 6 days when pneumonia also developed on the left side (**B**), and after *Stenotrophomonas maltophilia* was identified and treated with sulfamethoxazole/trimethoprim, a regression of lung infiltrates was observed (**C**).

On admission, the patient was hemodynamically unstable, with an arterial blood pressure of 80/50 mmHg, a pulse of 125 beats/min, and tachypneic with a frequency of >40 breaths/minute with oxygen saturation of 45% on room air. The leukocyte count was 5.3 × 10^9^/L, platelet count was 138 × 10^9^/L, and C-reactive protein level was 523 mg/L (Supplemental File S1). Arterial blood gas (ABG) analysis with a fraction of inspired oxygen (FiO_2_) of 0.8 revealed a pH of 7.22, PaCO_2_ level of 7.22 kPa, PaO_2_ level of 6.8 kPa, and bicarbonate concentration of 19 mmol/L. His urine output was normal (300 mL/h). Early vasopressor therapy (noradrenaline 10–20 mL/h), oxygen therapy, and analgosedation with midazolam and fentanyl were initiated. The patient was intubated and mechanically ventilated with positive end-expiratory pressure (PEEP) of 10 cmH_2_O with a FiO_2_ of 0.6–0.8 in controlled mechanical ventilation (CMV) mode. Purulent secretions were drained from the thoracic drain. An emergency thoracic CT was performed (Figure 1B and Figure 2A) and revealed extensive right-sided pneumonia of the lower and middle lung lobes and a minimal effusion of 1 cm. After microbiology samples were taken, empirical broad-spectrum antimicrobial therapy consisting of cefepime (2 g every 12 h) and levofloxacin (500 mg every 12 h) was initiated. Due to severe hemodynamic instability, analgosedation was discontinued.

The patient’s respiration did not improve within the first 2 days. Linezolid (600 mg every 12 h) and meropenem (1 g every 24 h) replaced the previous antibiotics on day 3. He was febrile (38.9 °C), unconscious on day 4, and was still ventilated with PEEP of 10 cmH_2_O with a FiO_2_ of 0.6 to obtain oxygen saturation >90%. Tracheal aspirate and bronchoalveolar lavage (BAL) samples did not confirm pathogenic bacteria. Due to paroxysmal atrial fibrillation, a cardiologist was consulted, who suggested IV propafenone therapy. On day 6, the patient was agitated; he was unconscious, unable to communicate, and breathed in synchronized intermittent mandatory ventilation (SIMV) mode. Upon auscultation, bronchial whistling and coarse rales were audible. The abdomen was soft, and peristalsis was audible. With propafenone therapy, the patient was in sinus rhythm with a frequency of 60 beats/min.

On day 8, due to the patient’s restlessness and inability to synchronize with the ventilator, a continuous infusion of dexmedetomidine was introduced, followed by a continuous infusion of midazolam and fentanyl due to extremely difficult ventilation with high oxygen concentrations and respiratory pressures (Paw greater than 30). In the intervals when interventions such as physical therapy and nursing interventions were performed, this asynchrony was significant. To optimize the patient’s care and comfort, he was relaxed using bolus doses of rocuronium. Between interventions, neuromuscular blockade was not required, and ventilation pressures were lower. A control CT of the thorax was performed, which revealed deterioration in terms of the progression of the inflammatory infiltrate to the right lung lobe (Figure 2B, Video S1). A thoracic surgeon was consulted several times.

He was hemodynamically stable during the day, with a decline in inflammatory parameters (CRP 207 mg/L, PCT 1.53 ng/mL) and with the presence of thrombocytopenia (platelet count 68 × 10^9^/L) (Table 1). He regained consciousness and was successfully weaned from the ventilator on day 10. Oxygenation was maintained with a Hudson face mask with a moderate O_2_ flow of about 10 L, which was gradually reduced. On physical examination, he still had prolonged expiration and crackles on the right side of the lung. All microbiological samples taken from the lungs remained sterile despite purulent secretions from the thoracic drain.

On day 15, a bronchoscopy with microbiology sampling was performed. After *S. maltophilia* was identified from the bronchoalveolar lavage, he was treated with sulfamethoxazole/trimethoprim. His white blood cell count was 16.3 × 10^9^/L, platelet count was 361 × 10^9^/L and C-reactive protein level was 203 mg/L (Table S1).

On day 18, the patient was awake, conscious, oriented, and breathing spontaneously via face mask, and his SpO_2_ was 95% (Supplemental File S1). The patient’s clinical status and chest radiograph both improved, and the thoracic drain was removed. The same day after the drain was removed, SpO_2_ decreased with silent breathing sounds apically on the right lung. Pneumothorax was suspected and confirmed after a chest radiograph. Re-thoracentesis was performed (Figure 2C, Supplemental File S1).

**Figure 3:**
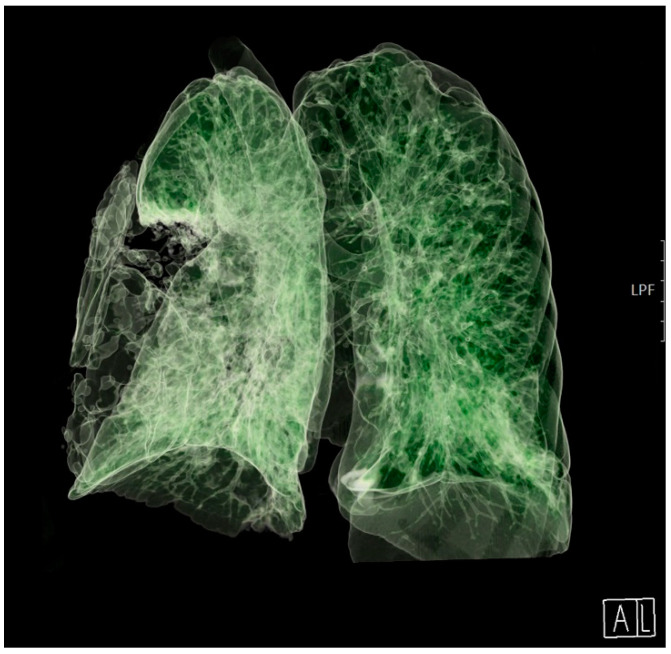
A 3D computed tomography scan of the lungs 25 days after admission to the Intensive Care Unit. A: anterior, L: lateral.

The patient’s clinical condition improved in the following days, and he continued to breathe spontaneously with oxygenation. The control chest radiograph of the lung revealed complete expansion of the lung parenchyma with lung infiltrates on the medial and lower lung lobes. The patient’s general condition gradually improved, and he was transferred to the ward on day 19 after ICU admission with supplemental oxygenation of 4 L O_2_/min via the nasal catheter and frequency of 18–20 breaths/minute with adequate oxygen saturation of 95–97%. The thoracic drain was removed on the ward (Figure 3).

FOLLOW UP. After the surgical treatment was completed, the patient’s medical therapy continued at the Department of Pulmonary Diseases. Sulfamethoxazole/trimethoprim was continued for a total of 14 days. He stopped smoking. Breathing problems, especially productive cough, mild tachypnea, and shortness of breath, lasted 3 months after the end of the ICU treatment.

Spirometry that was performed 6 months later. It confirmed that the patient’s FEV1 was 102%; FVC was 99%, while carbon monoxide (CO) diffusion capacity was reduced to 59%, and the diffusion coefficient was 56%. The proportion of residual volume (RV) was 47% of the total lung capacity, indicating hyperinflation (Table 1).

Blood gas analysis was also normal: pO_2_ 12.33 kPa; pCO_2_ 4.77 kPa; HCO_3_ 22 mmol/L; pH 7.407; SpO_2_ 97%.

**Figure 4:**
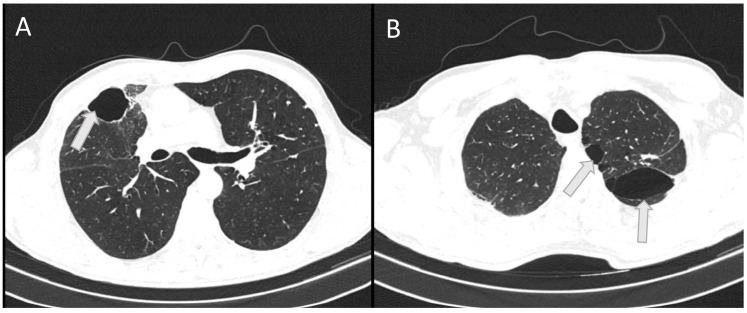
The horizontal CT scan section at the level of the 6th thoracic vertebra (**A**) and at the level of the 3rd thoracic vertebra (**B**) showing residual bullous changes (arrows) 6 months after severe chest injury with bilateral pneumonia in a 53-year-old patient.

On this outpatient examination, a CT scan of the lung was performed, which confirmed bullous changes in the left side of the lung (Figure 4). Subjectively, he had discomfort when breathing and pain in the right chest. He complained about poor physical condition. His medical therapy prescribed by a pulmonologist was ipratropium bromide (Atrovent N, Boehringer Ingelheim Pharma GmbH & Co., Ingelheim, Germany) 3 × 2 breaths.

## 3. Discussion

In this case report, we presented initial ventilation and supportive therapy in a patient with severe respiratory insufficiency after a blunt chest injury. The problem in treatment was the identification of the causative agent that was not confirmed via early microbiological diagnostics. Since V/Q mismatch was a consequence of lung injury, the treatment was focused on immediate management of the underlying condition itself, as well as simultaneous symptomatic treatment. The principles of management of hypoxemia include securing the airway, increasing inspired oxygen, identifying and treating the pathogen bacteria, removing secretions with intermittent bronchoscopies, and including respiratory support if necessary. A correction of hypoxemia, meticulous intravenous fluid management, and other supportive measures are crucial for improving the treatment outcome of patients with pneumonia [9].

Pneumonia due to *S. maltophilia* is not common, although according to statistics from large databases, the frequency of this infection has been increasing in recent decades, which is associated with the increased use of antibiotics and comorbidity of patients. According to a study by Duan and colleagues in a large academic hospital in China, out of 93 positive samples for *S. maltophilia*, underlying diseases were registered in 86 patients. A consistent characteristic of >90% of the strains is biofilm formation [10]. Sputum analysis in the U.S. studies in the period from 1995 to 2008 confirmed an increase in the prevalence of *S. maltophilia* from 6.7% to 12.0%. Bacterial isolation was significantly associated with patients who had a forced expiratory volume in 1 s <40%. According to the analysis of the SENTRY Antimicrobial Surveillance Program among pediatric patients, *S. maltophilia* was among the 15 top pathogens isolated from the Americas but not from Europe [11]. In our ICU, *S. maltophilia* is also very rare, with only two cases in a year, with a time gap of 6 months between two admissions.

The treatment of this pathogen should be considered in cases of pneumonia with confirmed *S. maltophilia* in sputum cultures after broad-spectrum antibiotics were given for a longer time period and in patients with immunodeficiency with a high SOFA score [12]. Since *S. maltophilia* is intrinsically resistant to several types of antibiotics, it is potentially difficult to treat. In a retrospective study, Shah and co-authors demonstrated that combination therapy had similar rates of clinical efficacy and resistance development compared to monotherapy for *S. maltophilia* pneumonia [13]. The authors have observed that 30-day mortality was greater with combination therapy [13]. Trimethoprim–sulfamethoxazole remains the drug of choice, although in vitro studies indicate that ticarcillin–clavulanic acid, minocycline, some of the new fluoroquinolones, and tigecycline may be useful agents [14].

This patient clearly had a “two-hit” insult. We assume that the initial infection with septic shock was caused by another causative agent, which responded well to the empirical therapy started. It is possible that *S. maltophilia* was in the injured part of the lung from the beginning of the injury. Our patient was a smoker, so it is possible that he had undetected structural lung disease at the time of injury [10,15]. In addition, he was a worker in a shipyard, a humid environment that favored the growth of *S. maltophilia*. These are the factors that favor the growth of *S. maltophilia*, which grows well in moist habitats. However, we could not prove it in the aspirates from the left lung until the collapsed part of the lung was not ventilated. Since *S. maltophilia* is a Gram-negative obligate aerobe, in ventilated lungs, it had better conditions for growth, and a second-hit insult occurred with an increase in inflammatory parameters. The introduction of targeted therapy with trimethoprim–sulfamethoxazole led to the regression of the inflammatory parameters and lung infiltrates (Supplementary File S1).

In addition to the infection treatment, maintaining adequate oxygenation was a significant clinical problem due to the large areas of crushed lung tissue that were not ventilated. An increase in inspired oxygen (FiO_2_ 100%) leads to an increase in alveolar oxygen tension (P_A_O_2_) to greater than 100 mmHg, even in lung regions with very low alveolar ventilation. On the contrary, regions of the intra-pulmonary shunt are not ventilated, and those regions have an oxygen content equal to the mixed venous blood [16].

In this patient, the maintenance of satisfactory saturation was achieved by a combination of high oxygen concentrations and moderate ventilation pressures in the most severe period of inflammation with bilateral pulmonary infiltrates that were gradually reduced. Barotrauma inevitably occurs with prolonged mechanical ventilation with high pressures [17,18]. We tried to reduce it by avoiding PEEP greater than 10 mmHg with a higher percentage of oxygen. In this patient, we personalized the ventilation according to the findings of the CT scan. This enabled dynamic adjustment of PEEP to the lowest possible values, according to current clinical indications. Maintaining ventilation at the lowest possible pressures was necessary to avoid lung barotrauma. Additionally, according to the findings of the CT scan, we could decide to perform fiberoptic bronchoscopy and targeted bronchoalveolar lavage. The aim of this procedure was to simultaneously evacuate secretions and blood and establish the patency of the bronchi in the injured and inflamed area of the lung. Finally, targeted bronchoscopic sampling from perfused and poorly ventilated areas of the lungs made it possible to take samples for microbiological analysis and causal treatment of the causative agent.

High ventilation pressures can improve oxygenation but may increase the risk of barotrauma in the healthy lung. Due to lower resistance, a larger volume of inhaled gas will be directed to healthy areas of the lungs [17]. This is the reason why the strongest bullous changes after mechanical ventilation in our patient were found on the follow-up CT after discharge in the lung areas that were less affected by trauma and inflammation, i.e., in the upper left lung lobe.

In this patient, we considered other modalities that could improve oxygenation and CO_2_ elimination. During the first few days, the patient had occasional episodes of asynchrony, especially during nursing interventions and physical therapy. We solved them by intermittent bolus administration of rocuronium. Continuous muscle relaxation was not necessary because we were able to achieve satisfactory oxygenation in the patient with sedation and analgesia.

Prone positioning was difficult due to thoracic drainage, as the patient had a drain on the right side and due to his initial hemodynamic instability. When the patient became hemodynamically stable, physical therapy along with ventilator respiratory therapy was started.

We also considered extracorporeal membrane oxygenation (ECMO). During the period when the patient’s ventilation was at its worst, he was thrombocytopenic, so we tried to achieve oxygenation without ECMO. Considering that ECMO has its own complications, including bleeding and infections, and that it requires anticoagulation, we avoided the application of ECMO with the previously mentioned methods of mechanical ventilation.

Barotrauma can lead to the destruction of alveoli, parenchymal bleeding, and acute infiltration during the acute injury itself [18]. After the resolution of the inflammatory reaction, due to the resulting residual fibrosis, these affected alveoli will not contribute to gas exchange. Impaired perfusion can also be expected in tissues after severe inflammation. The consequence of post-traumatic and post-inflammatory events in our patient was an increase in non-functional residual lung volume, with the development of emphysema and decreased gas exchange function. A residual volume of 40% of the total lung volume measured in our patient is a poor prognostic sign [19].

Reanalyzing this case, a shortcoming of this treatment could be that a viral respiratory panel was not analyzed on arrival. Although the treatment for most viruses is symptomatic, the recognition of the virus as the causative agent may have shortened the duration of antibiotic therapy. Another shortcoming is that we could not perform respiratory mechanics measurements in the acute phase because the patient had chest drainage. Therefore, we could only record the default values on the ventilator.

In conclusion, both ventilatory and antibiotic therapy were needed to improve the patient’s oxygenation and outcome after chest trauma with V/Q mismatch and pneumonia due to the resistant *S. maltophilia*. It is important to maintain a high index of suspicion for resistant organisms such as *S. maltophilia* infection, especially in patients who are not responding to empiric broad-spectrum antibiotics [20]. Careful balancing between the oxygen ratio and ventilation pressure is needed to maintain oxygenation. Such an approach may alleviate the consequences of lung trauma with severe pulmonary infections.

## Figures and Tables

**Table 1:** CO diffusion capacity after the treatment in a 53-year-old male patient after severe blunt chest trauma 6 months after the injury.

Parameter	Unit	Predicted	Best	%(Best/Predicted)
DLCO_SB	mmol (min·kPa)	9.25	5.50	59
KCO_SB	mmol (min·kPa·L)	1.44	1.20	83
VA_SB	L	6.27	4.6	73
Hemoglobin	g/dL		14.6	
DLCOcSB	mmol (min·kPa)	9.25	5.5	59
KCOc_SB	mmol (min·kPa·L)	1.44	1.2	83
BHT	s		11.00	
VIN_SB	L	4.17	2.5	60
TLC_SB	L	6.42	4.72	73
FRC_SB	L	3.34	2.93	88
ERV_SB	L	2.15	2.23	104
RV%TLC_SB	%	35	47	136

Note: DLCO, diffusing capacity of the lung for CO; SB, single breath; KCO, transfer coefficient of the lung for carbon monoxide; VA, alveolar volume; DLCOcSB, carbon monoxide diffusion capacity; KCOc, carbon monoxide transfer coefficient; BHT, breath holding time; VIN, volume inspired; TLC, total lung capacity; FRC, functional residual capacity; ERV, expiratory reserve volume; RV%TLC, residual volume to total lung capacity ratio.

## Data Availability

All data significant for this case report are published in this paper or in the Supplemental File.

## Supplementary Materials

The following supporting information can be downloaded at: https://www.mdpi.com/article/10.3390/jcm13030871/s1, Supplemental File S1. Table S1: Laboratory parameters in the patient with severe lung injury and pneumonia caused by *Stenotrophomonas maltophilia*; Supplemental Figures S1–S10 arranged by dates after admission; Video S1: Lung CT after 8 days showing bilateral pulmonary infiltrates.
