# Supplementary material for: Ventilation Management in a Patient with Ventilation–Perfusion Mismatch in the Early Phase of Lung Injury and during the Recovery
Source: J Clin Med. 2024 Feb 2;13(3):871. doi: 10.3390/jcm13030871 (PMC10856224; doi:10.3390/jcm13030871)
Supplement: Supplementary file 1 [file jcm-13-00871-s001.zip › Cicvaric_suppl.file1.pdf]

Journal of
Clinical Medicine

MDPI

Supplemetal File S1

Ventilation Management in a Patient with Ventilation–Perfusion Mismatch in the Early Phase of Lung Injury and during the Recovery

Ana Cicvarić [1,2,*], Josipa Glavaš Tahtler [1,2], Tajana Turk [1,3], Sanda Škrinjarić-Cincar [4], Despoina Koulenti [5,6], Nenad Nešković [1,2], Mia Edl [1] and Slavica Kvolik [1,2,*]

[1] Faculty of Medicine, Josip Juraj Strossmayer University of Osijek, 31000 Osijek, Croatia; josipa.glavastahtler@kbco.hr (J.G.T.); turk.tajana@kbco.hr (T.T.); neskovic@mefos.hr (N.N.); medl@mefos.hr (M.E.)
[2] Department of Anesthesiology, Resuscitation and Intensive Care, Osijek University Hospital, 31000 Osijek, Croatia
[3] Department of Radiology, Osijek University Hospital, 31000 Osijek, Croatia
[4] Department of Pulmonology, Osijek University Hospital, 31000 Osijek, Croatia
[5] 2nd Critical Care Department, Attikon University Hospital, 15772 Athens, Greece; deskogr@yahoo.gr
[6] UQ Centre for Clinical Research, Faculty of Medicine, The University of Queensland, Brisbane 4029, Australia
* Correspondence: annikaa.dv@gmail.com (A.C.); skvolik@mefos.hr (S.K.); Tel.: +385-31-511-511 (A.C.); +385-31-511-502 (S.K.)

Day	1	2	3	4	5	6	7	8	9	10	11	13	14	15	16	17	18	19	20	21	22	23
**WBC(x10^9/L)**	3.6	5.3	17.6	30.2	25.4	18.9	16.7	18.3	23.6	20.3	18.6	19.1	19.4	16.7	18.05	16.3	12.8	12.1	15.8	13.4	12.7	13
**CRP(mg/L)**	523.9	388.7	428		246.4	218.5	147.4	207.9	153.5	228.7		145.5	164.5	191.5		203	183.9	188.4	192.1	190	222.1	159.2
**PCT(ng/mL)**		35.88	44.55	28.36	13.97	7.32	3.43	1.53	0.93	0.65												
**RBC(x10^{12}/L)**	4.12	3.67	3.86	3.29	3.54	3.47	3.34	3.23	3.15	2.99	2.63	2.88	2.71	2.87	3.03	2.85	2.68	2.98	2.92	3.14	2.96	2.85
**Hb(g/L)**	132	118	125	105	114	111	107	103	102	96	86	92	87	92	93	89	85	93	91	96	89	87
**Hct(L/L)**	0.384	0.343	0.363	0.315	0.34	0.335	0.326	0.322	0.312	0.298	0.255	0.276	0.262	0.27	0.289	0.275	0.26	0.28	0.274	0.299	0.284	0.274
**PLT(x10^9/L)**	180	138	158	121	105	67	60	68	135	190	251	335	378	349	362	361	316	295	280	244	309	312
**Fibrinogen (g/L)**									3.9											4.4		
**Na(mmol/L)**	135	138	145	145	147	152	151	149	148	148	143	142	143	143	141	141	141	142	139	142	140	138
**K(mmol/L)**	4.8	4.3	4.7	4	4.2	4.2	3.9	3.7	3.4	3.8	3.6	4	3.8	3.8	4.1	4.1	3.9	3.9	4	4	4.4	4.4
**Albumin (g/L)**			21.8	18.8	23.9			18.6	18.5			19.9				22.4				23.4		

Table S1 Laboratory indicators in the patient with severe lung injury and pneumonia caused by *Stenotrophomonas maltophilia*.

Note. WBC-white blood cell; CRP-C-reactive protein; PCT-procalcitonin; RBC-red blood cell; Hb-hemoglobin; Hct-hematocrit; PLT-platelet count; PT-prothrombin time; INR-international normalized ratio; aPTT-activated partial thromboplastin time; Na-sodium; K-potassium

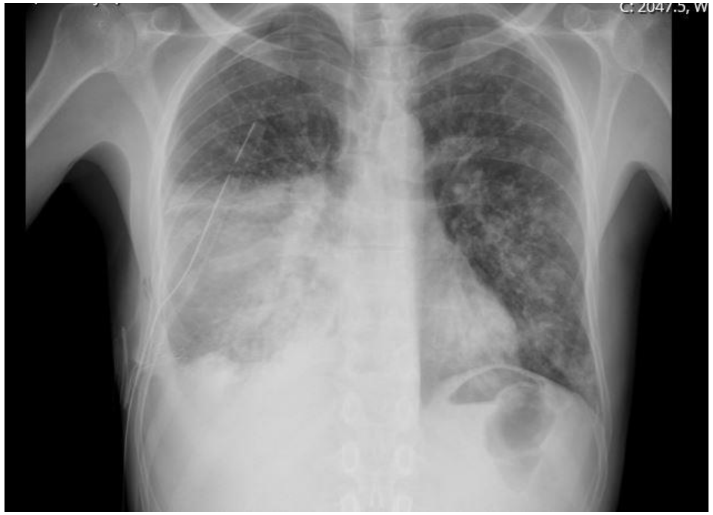

Figure S1. Day1_Dec.17

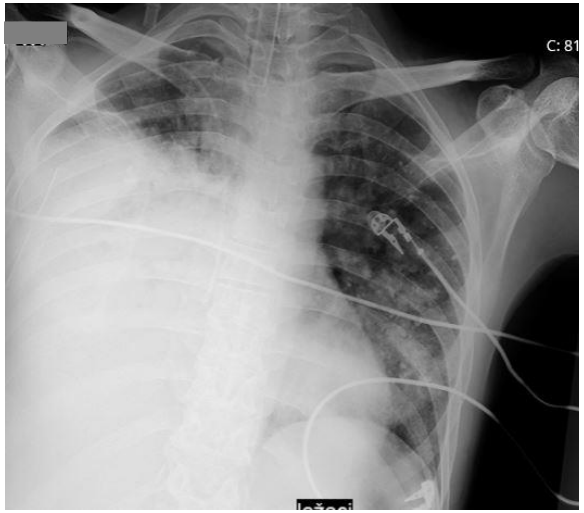

Figure S2. Day3_Dec19

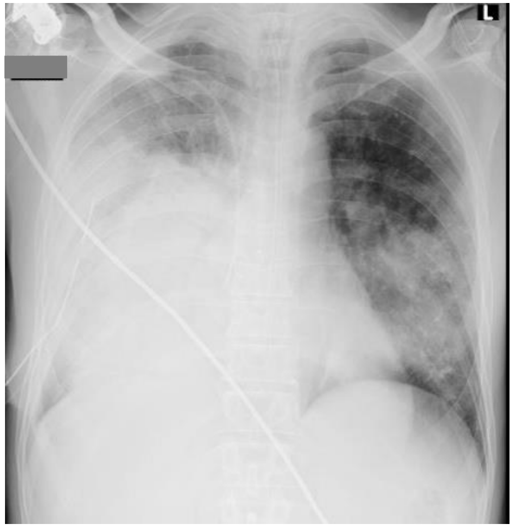

Figure S3. Day4_Dec20

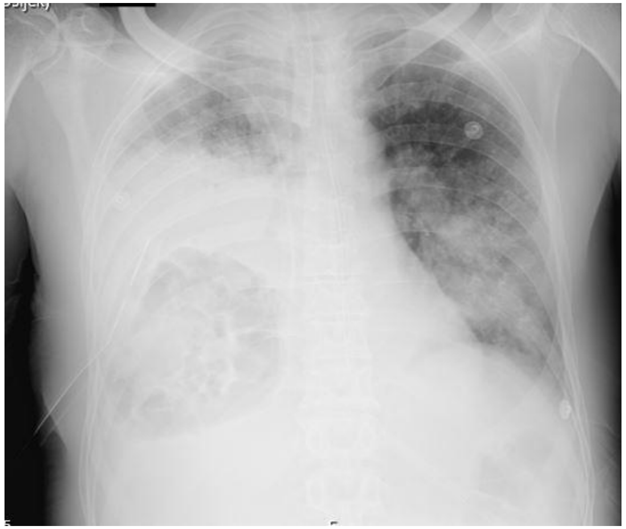

Figure S4. Day7_Dec24.

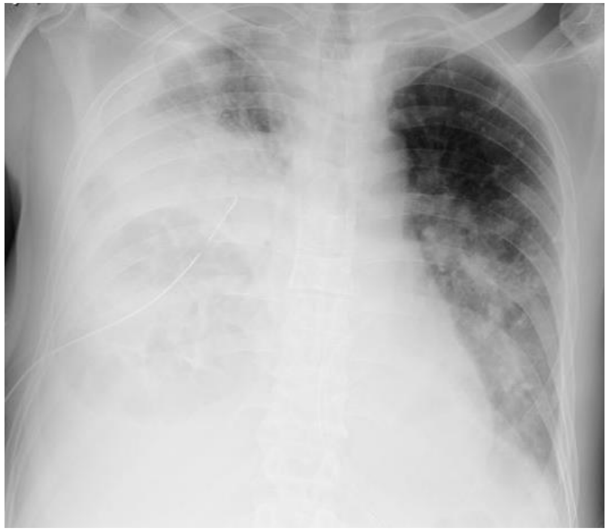

Figure S5. Day10_Dec26

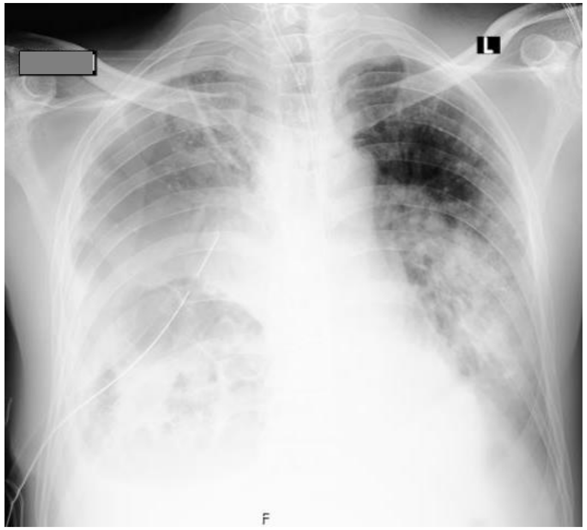

Figure S6. Day12_Dec28

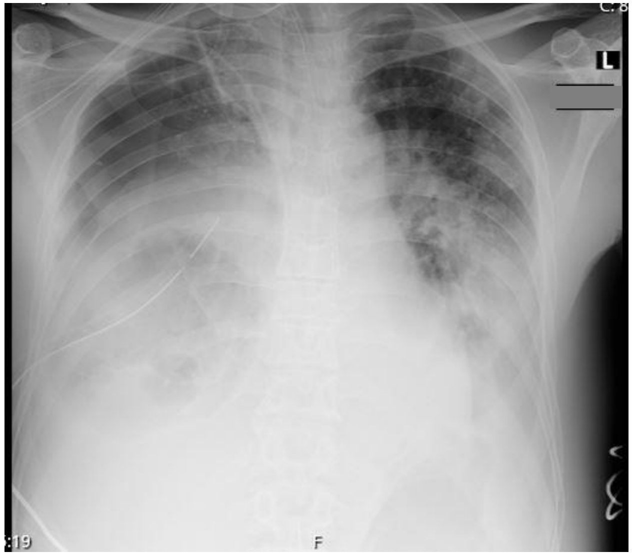

Figure S7. Day14_Dec30

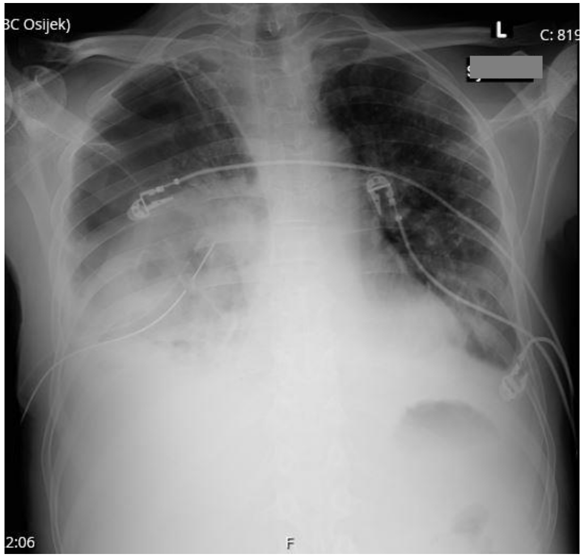

Figure S8. Day17_Jan02.

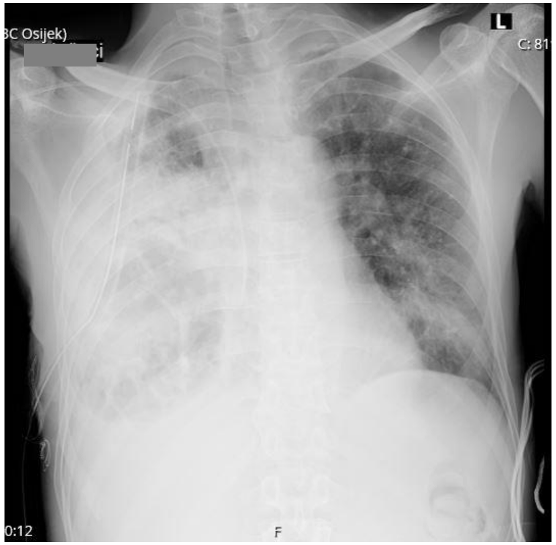

Figure S9. Day19.Jan04

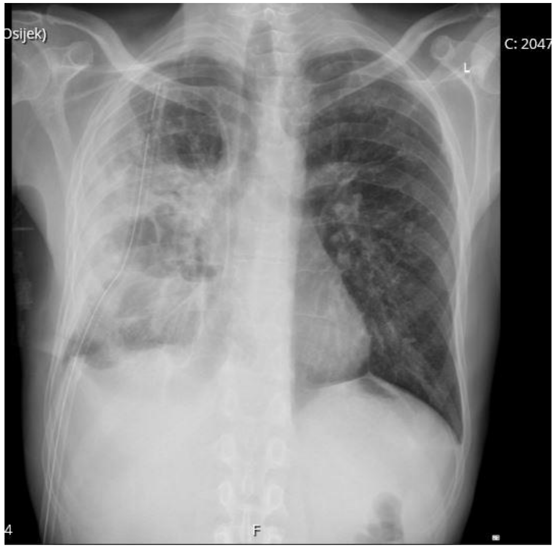

Figure S10. Day29_Jan14
